# Connecting Through Conversation: A Novel Video-Feedback Intervention to Enhance Long-Term Care Aides’ Person-Centred Dementia Communication

**DOI:** 10.1177/23337214221101266

**Published:** 2022-05-14

**Authors:** Deanne J. O’Rourke, Michelle M. Lobchuk, Genevieve N. Thompson, Christina Lengyel

**Affiliations:** 1 College of Nursing, Rady Faculty of Health Sciences, 423134 University of Manitoba, Winnipeg, MB, Canada; 2 Department of Food and Human Nutritional Sciences, Faculty of Agricultural & Food Sciences, 98691 University of Manitoba, Winnipeg, MB, Canada

**Keywords:** person-centered, communication, dementia, video feedback, nursing home

## Abstract

**Objective:**

To pilot test a novel communication intervention incorporating a video-feedback component on the person-centred dementia communication skills of long-term care aides.

**Methods:**

Effectiveness was assessed using a single group pre-test/post-test design. 11 care aide-resident dyads participated in the study. Objective outcomes included provider statements demonstrating linguistic (i.e., reciprocity, clarity/coherence, and continuity categories) and relational elements of person-centred dementia communication, measured via video-recorded observations of usual care interactions. Subjective outcomes of care aide communication confidence/competence, satisfaction with the resident relationship, relationship closeness, and self-reflection at work were measured using self-report questionnaires.

**Results:**

In respect to observed person-centred dementia communication skills, there was an increase in the use of linguistic statements in the reciprocity and continuity categories, as well as total linguistic statements overall. Relational statements and overall person-centred dementia communication (i.e., linguistic plus relational strategies) increased. Care aide-reported communication confidence and competence, relationship closeness with the resident, and self-reflection at work also increased after the communication intervention.

**Discussion:**

The communication intervention showed promise as an effective approach to enhance person-centred dementia communication behaviours in care aides. These results support undertaking a larger trial to examine the intervention’s effectiveness more fulsomely.

## Introduction

Person-centred care has been promoted widely as the gold standard of care for older adults (American Geriatrics Society, 2016). Person-centred care in long-term care (LTC) refers to a philosophy that emphasizes relationship and interdependency as well as the concepts of individualism, holism, respect, and empowerment of those that live, work or are otherwise a part of a LTC community (Harding, Wait, & Scutton, 2015). These central attributes of a person-centred philosophy are fundamentally integrated in day-to-day communication and interactions between care providers and residents (Kitwood, 1997). Research shows that LTC residents react more positively (Savundranayagam et al., 2016), experience enhanced mood and affect (McGilton, Sidiani, Boscart, Guruge, & Brown, 2012) and report higher levels of well-being (Custers et al., 2011) when care providers demonstrate effective relational behaviours during interactions.

Despite these cited benefits, for care providers working within a demanding LTC environment, recognizing, and responding to residents’ relational needs are often missed (Savundranayagam, 2014) or sacrificed in exchange for expediency (Knopp-Sihota et al., 2015). Up to 80% of communication by care providers with persons experiencing dementia is task-focused (Wilson, Rochon, Leonard, & Mihailidis, 2013). Excessive or exclusive task-based communication in this manner diminishes the opportunity to acknowledge the unique value and contribution of the person as a communication partner (Williams, 2013). It has been recognized that there is a need to augment care providers’ communication skills to promote interactions of a relational nature in concert with person-centred principles (Carpiac-Claver & Levy-Storms, 2007). As such, an opportunity exists to enhance the quality of interaction between care providers and residents by embedding relational communication strategies in daily care activities.

Interventions to enhance person-centred communication skills in care providers have begun to emerge in the LTC literature; however, a significant limitation of strategies to-date relates to the lack of attention to the self-reflective aspect of learning. This is relevant to person-centred culture change, as realization of care providers’ outward person-centred behaviours requires a turning inward to reflect upon personal beliefs and values about one’s caregiving philosophy (Viau-Guay, 2013).

A promising self-reflective technique that has potential to improve person-centred communication skills is video-feedback (VF) (Fukkink et al., 2011). VF is a learning technique in which individuals watch video-recorded examples of their own performance in a real-world or simulated encounter (Williams & Gallinat, 2011). Only one study was found that tested its use to promote person-centred approaches in LTC (Coleman & Medvene, 2013). However, emerging evidence in intellectual disability suggest that VF can improve care providers’ individualized care approaches (Embregts, 2002; Zijlmans, Embregts, Gerits, & Derksen, 2011), promote a shift in perspective-taking that allows the care provider to understand and imagine the other’s viewpoint, and facilitate values-based changes in their performance through self-reflection (James et al., 2016).

Data indicates that nearly half the residents in LTC experience limited or no social engagement (CIHI, 2021). Since most personal care in LTC is provided by care aides (Estabrooks et al., 2014) there is an opportunity to foster learning and self-reflective opportunities to enhance the nature and quality of their person-centered relationships with residents. Given that up to 63% of LTC residents have a formal diagnosis of dementia (CIHI, 2021; Harris-Kojetin et al., 2019) and 83% have some degree of cognitive impairment (CIHI, 2021), communication competence requires a specific skill set and approach (Downs & Collins, 2015). As such, this intervention aimed to enhance the quality of the relationship and communication between care aides and LTC residents experiencing dementia.

## Methods

### Study objective

The primary objective was to pilot test an intervention incorporating VF on long-term care aides’ person-centred dementia communication (PCDC) behaviours and perceived quality of relationship with residents who have mild to moderate dementia. For this initial pilot test, a decision was made to focus on residents with mild to moderate dementia as there was a higher likelihood of residents retaining verbal communication skills in those stages. As such, it was hypothesized that the care aides could engage the residents in conversation and utilize their PCDC skills. Key definitions and concepts related to this study are outlined in Table 1.

**Table 1.** Key Concepts and Definitions.

Concept	Definition
Long-term care	Residential facilities that provide 24-hour professional nursing care and supervision in a protective, supportive environment for people who have complex care needs and can no longer be cared for in their own homes
Person-centred care	“Person-centred care means that the individuals’ values and preferences are elicited and…guide all aspects of their health care, supporting their realistic health and life goals. Person-centred care is achieved through a dynamic relationship among individuals, others who are important to them, and all relevant providers. This collaboration informs decision-making to the extent that the individual desires” (AGS, 2016, p. 16).
Person-centred communication	Communication approaches that support the overarching principles of person-centred care, namely value and respect for the person, promotion of an individualized approach to care and understanding of the person’s perspective within a relationship context (Brooker, 2007; Kitwood, 1997)
Person-centred dementia communication	Refers to person-centred communication approaches for individuals with dementia, defined as consisting of both linguistic (language-based) and relational (person-centred) elements (Kitwood, 1997; Savundranayagam & Moore-Nielsen, 2015)
Care aides	Unregulated healthcare workers who provide direct care (e.g., bathing, dressing, oral care, continence, meal-time assistance, mobility/ambulation, etc.) to residents in long-term care homes (Estabrooks et al., 2014)
Video feedback	A self-reflective technique where learners watch video-recorded examples of their own performance in a real-world or simulated encounter (Williams & Gallinat, 2011). Within this study, video feedback was employed within a one-on-one setting involving the learner and a facilitator.

### Study design

The study was conducted between January and May 2019 in an urban LTC home in Manitoba, Canada after receiving ethical approval from the Education/Nursing Research Ethics Board at the University of Manitoba. A single group pre-test/post-test design was used to observe for intra-participant differences in the care aides’ response to the intervention. The timeline for the study is outlined in the supplemental materials.

The baseline and post-intervention videos captured usual care interactions and were recorded using a portable electronic device (Microsoft® Surface). Baseline and post-intervention videos captured the same type of care encounter, e.g., if morning care was recorded for the baseline video, then morning care was also captured for the post-intervention video. To reduce anxiety and potential observation bias from the video-recording, the first author reviewed the purpose and video procedure with the staff and resident participants prior to the recorded care episode. Assent to proceed with recording was obtained in every instance.

### Participants

As the intervention had not been employed within this research context, a power analysis could not be conducted, nor an effect size determined. Thus, in consultation with a biostatistician, based on the pilot study design and aim to trial the intervention with a small, representative group of care aides in LTC, the sample size target was between 10-20 participants. Inclusion criteria for care aide participants were: provided regular care and/or assistance to residents, held a position of either full-time or part-time status, worked either day or evening shifts, and were able to speak and read English. Resident inclusion criteria were: had a diagnosis of dementia (any subtype), had mild to moderate stage of dementia [defined as a current Resident Assessment Instrument (RAI) Cognitive Performance Scale (CPS) score of 1, 2 or 3 (Morris et al., 1994)], and provided informed consent or had a substitute decision-maker (SDM) who provided consent.

### Recruitment

General information pertaining to the study was shared with staff, residents, and families by means of study invitation letters, posters, and staff meetings. Interested care aides were approached by the first author to gain written consent. Care aides were asked to confidentially identify potential resident partners and seal the list in an envelope. The sealed envelopes were given to a nurse manager who evaluated the identified residents as to the inclusion criteria. If the resident met the study criteria, the manager contacted the resident or their SDM to inquire as to their interest in the study and request consent to forward their contact information to the first author. A meeting was then arranged with the resident or SDM to gain written consent.

### Description of the intervention

The PCDC intervention was comprised of two components: a group educational workshop followed by a one-on-one VF session. Development of the education session content incorporated the theoretical and empirical evidence in relation to person-centred care and PCDC. The three-hour education session facilitated by the first author addressed the cognitive and behavioural components of learning PCDC skills (McGilton et al., 2009) using reflective techniques and activities, small and large group discussion, analysis and critique of communication examples, and role play. The session plan is provided in the supplemental materials.

The individual VF session involved a single 30–45-minute session between the first author and each care aide two to three weeks after completion of the education session. The care aides reviewed their baseline video of the interaction with their resident partner. To stimulate active and self-reflective learning (Williams & Gallinat, 2011), the care aides were asked to note displayed linguistic and relational PCDC behaviours using the same checklist reviewed in the education session. The care aides were then asked to verbally share their reflections in relation to displayed PCDC skills, and subsequently any missed opportunities for a person-centred response that they noted. Any additional feedback was offered by the facilitator for the purposes of coaching and teaching, and the participant was asked to self-identify communication goals they would like to focus on for improvement over the coming weeks.

### Outcome measures

#### Linguistic PCDC elements

The categorization of PCDC linguistic strategies and related coding system developed by Savundranayagam and Moore-Nielsen (2015) was used to observe for any linguistic changes in PCDC. The coding system involves examining care provider utterances for evidence of any of the 21 linguistic strategies and recording an absolute count of each linguistic strategy noted within the care interaction. The 21 linguistic strategies were categorized according to their relative communication goal: 1) reciprocity, to encourage two-conversation; 2) clarity/coherence, to promote clear understanding and communication; and 3) continuity, to support the resident to continue the conversation or activity. To demonstrate, an example of a reciprocity strategy would be to facilitate taking turns speaking during a conversation and giving time for the resident to respond. An approach that promotes clarity/coherence would be to confirm understanding of a resident’s statement by asking for clarification. Lastly, by placing emphasis on the noteworthiness of a resident’s prior statement would be an example of a continuity strategy with the aim to encourage ongoing participation in the conversation. In respect to reliability of the PCDC linguist coding system, the originating authors reported a 91% agreement analysis between two trained researchers during independent coding of transcripts (Savundranayagam & Moore-Nielsen, 2015).

#### Relational PCDC elements

The relational elements of PCDC were evaluated using a measurement approach described in the empirical literature (Savundranayagam, 2014; Savundranayagam & Moore-Nielsen, 2015; Savundranayagam et al., 2016). Aligning with Kitwood’s four indicators of positive person work relevant to PCDC (Kitwood, 1997), this coding scheme identifies the presence of recognition, negotiation, validation, and facilitation/collaboration within care provider interactions with LTC residents during care activities. The coding approach involves examining care provider utterances for evidence of any of the four relational strategies and recording an absolute count of each noted within the care interaction. An example of a statement of recognition would be to acknowledge the resident as a person, by their name or in a unique way. To negotiate means using statements or approaches with the intent to consult about the resident’s preferences, desires, and needs. A validation strategy uses empathy to gain a sense of what the resident may be experiencing. And lastly, an example of a facilitation/collaboration approach might engage the resident in a shared task with a defined goal.

For reliability testing of the relational PCDC coding system, agreement analysis of two trained researchers was reported to be 91% for recognition, 92% for negotiation, 85% for validation, and 84% for facilitation/collaboration (Savundranayagam, 2014; Savundranayagam & Moore-Nielsen, 2015; Savundranayagam et al., 2016). Details of the linguistic and relational coding system, and steps taken to address rigor of coding in this study are outlined in the supplemental materials.

#### Competence and confidence

The care aides’ perceived competence and confidence in communicating with residents experiencing dementia was measured using the Providers’ Interactional Comfort Survey (Bowles et al., 2001). This six-item tool measures perceptions of provider competence, confidence, willingness, and scope of practice related to communication with patients/residents/clients. Total scores range from 0 to 60, with higher scores indicating increased competence and comfort in communicating with residents (Bowles et al. 2001). The scale has demonstrated acceptable internal consistency reliability in previous empirical work with care aides in the LTC setting (Cronbach’s alpha coefficient of 0.81) (McGilton, Irwin-Robinson, Boscart, & Spanjevic, 2006) and has shown sensitivity to a communication intervention (McGilton et al., 2010).

#### Relationship satisfaction

Self-reported relationship satisfaction with the resident was measured using the Personal Accomplishment subscale of the Maslach Burnout Inventory (Maslach & Jackson, 1981). It is comprised of eight items that describe feelings of success and achievement in relation to one’s work and provision of care/service to others. Total scores span from 0 to 48, with higher values suggestive of greater feelings of accomplishment. Internal consistency of the PA subscale was originally reported as 0.71 (Cronbach’s coefficient alpha) (Maslach & Jackson, 1981). Subsequently, across a wide range of samples and empirical studies, reliability coefficients have shown similar internal consistency for the PA subscale (Maslach et al., 2016).

#### Relationship closeness

Relationship closeness with the resident was measured using the Mutuality Scale, created by Archbold et al. (1990). This 15-item scale is comprised of four factors: shared pleasurable activities, shared values, love, and reciprocity (Archbold et al., 1990). The total scoring ranges from 0 to 60 with higher values indicating greater relationship mutuality. Initial testing and subsequent studies have reported high reliability of the MS with a Cronbach’s alpha of >0.90 (Archbold et al., 1990; Heliker & Nguyen, 2010; Lyons et al., 2007; Pucciarelli et al., 2016).

Additionally, global ratings of relationship closeness with the resident were collected using the Provider Close Visual Analogue Scale (VAS) (McGilton et al., 2010). The measurement tool is a 100 mm scale with anchors ‘*Not at all close provider-resident relationship’* and ‘*Very close provider-resident relationship’*. The Provider Close VAS has been used in previous study of provider-resident relationship closeness and has demonstrated acceptable test-retest reliability (r = 0.90) and responsiveness to change (McGilton et al., 2010).

#### Self-reflection

Self-reflection was measured using a global rating score created by the first author in response to the question ‘How often do you reflect upon (or think deeply) about your feelings and actions at work to help you understand the resident’s situation?’ The measurement tool is a VAS with anchors ‘*Never’* and ‘*All the time’*. Participants indicated their response to the question by drawing a vertical line across a 100 mm horizontal line (VAS). The score was determined by measuring in milometers from the far left anchor (e.g., ‘*Never’ response)* to the participants’ drawn line. Possible scores ranged from 0 (‘*Never’)* to 100 (‘*All the time’)*.

#### Co-variate indicators

For descriptive purposes and to assess for interaction effects, care aide (Table 2) and resident (Table 3) information was collected.

**Table 2.** Care Aide Characteristics.

	Mean (±SD)	Median (Range)	
Age* (years)	40.6 (9.02)	41.0 (24.0–52.0)	
Shifts worked in past 2 weeks	9.2 (0.87)	9.0 (8.0–10.0)	
How long working with resident partner* (months)	21.9 (23.74)	15.0 (3.5–84.0)	
Years worked on current floor/unit	3.1 (2.65)	2.0 (0.8–9.0)	
Years worked in LTC	10.7 (8.39)	9.0 (1.1–24.5)	
Years worked as HCA	10.6 (8.52)	9.0 (1.1–24.5)	
		n (%)
Gender		
• Female		9 (82%)
• Male		2 (18%)
First language		
• English		6 (55%)
• Other		5 (45%)
Country of birth		
• Canada		5 (45%)
• Other		6 (55%)
Highest level of education completed		
• College program/certificate		9 (82%)
• University degree		2 (18%)
Primary shift worked		
• Days		6 (55%)
• Evenings		5 (45%)
Current position		
• Full-time		7 (64%)
• Part-time		4 (36%)

*N* = 11; *N = 10.

**Table 3.** Resident Characteristics.

	Mean (±SD)	Median (Range)	
Age (years)	88.9 (8.57)	88.0 (74–103)	
Number of active medical diagnoses	7.4 (2.07)	7.0 (5–11)	
Number of medications (OTC and prescriptions)	9.8 (4.42)	9.0 (4–17)	
^a^Cognitive performance scale score	2.5 (0.71)	3.0 (1–3)	
^b^Index of social engagement score	3.0 (1.49)	2.5 (1–6)	
		n (%)
Sex		
• Female		9 (90%)
• Male		1 (10%)
Sub-type of dementia		
• Alzheimer		2 (20%)
• Vascular		2 (20%)
• Unknown		6 (60%)
Number of staff assist – pre-video		
• One		7 (70%)
• Two		3 (30%)
Number of staff assist – post-video		
• One		6 (60%)
• Two		4 (40%)

*N* = 10 (one resident acted as partner for two Health Care Aides).

^a^Cognitive Performance Scale range: 0–6.

^b^Index of Social Engagement range: 0–6.

### Statistical methods

A significance level of *p <* 0.1 was used for all statistical tests with the intent to detect clinically-relevant effects within a small sample (Shadish, Cook & Campbell, 2002). The Statistical Package for Social Sciences version 26 (IBM, 2019) was used to conduct the statistical testing. The statistical analysis plan was developed in consultation with a biostatistician and the steps are detailed below:

1. Missing data were addressed by averaging the other responses in the respective scale or measure.
2. The Kolmogorov-Smirnov and Shapiro-Wilk statistical tests and histogram charts were used to assess the data for normal distribution. If at least one of the statistical tests was significant at the 0.1 level and/or the histogram indicated normal distribution, the scores were considered normally distributed.
3. To observe for unadjusted intra-participant changes in outcomes between pre-intervention and post-intervention measures, paired samples t-tests were used for normally distributed data (17 outcome measures) and the Wilcoxon-Signed Rank tests were utilized for non-normal distributed data (one outcome measure).
4. To inform the regression analysis, independent samples testing was conducted using parametric (normally distributed data) and non-parametric (non-normal distributions) bivariate correlational tests to determine the presence of any relationship between the independent variables (i.e., 21 care aide and resident covariates in total) and outcome variables.
5. To explore intra- and inter-participant effects on the outcome variables of interest, univariate regression analysis was conducted using a repeated measures general linear model (GLM) procedure (Dobson & Barnett, 2008; Fitzmaurice et al., 2011). Covariates from the independent samples testing in Step Four that had a significant relationship with any of the outcome variables were included in the regression model (*n* = 13).
6. Using Cronbach’s alpha, reliability testing of the individual scale items [i.e., linguistic, relational, and overall PCDC communication skills, Provider Interaction Comfort Scale (PICS), Personal Accomplishment (PA), and Mutuality Scale (MS), was undertaken to inform the interpretation of results. A reliability estimate of 0.75 or greater was considered acceptable based on sample size guidelines for the Cronbach’s alpha test (Mohamad, Evi, & Nur, 2018).

## Results

### Description of the sample

The study sample consisted of 11 care aide-resident dyads. One resident acted as partner for two care aides; therefore, the total number of resident participants was 10. All care aides completed the study; however, one participant unexpectedly left their employment prior to the post-intervention video being taken. All other pre- and post-data were obtained from this participant. Characteristics of the care aide participants (*N* = 11) are outlined in Table 2 and residents (*N* = 10) in Table 3.

### Video characteristics

Twenty-one videos were obtained across 11 care aide-resident dyads. The length of the pre-intervention videos (*n* = 11) was 13.1 minutes (mean, SD = 5.09; range = 6.3–22.0) and the post-intervention video (*n* = 10) length was 12.4 minutes (mean, SD = 4.3; range = 6.0–19.5). The video recording occurred during morning care (*n* = 5), evening care (*n* = 5), or exercise sessions (*n* = 1). All video recordings occurred within a private setting, i.e., in the resident’s room or an exercise room.

### Main findings

Table 4 summarizes the results of the pre- and post-intervention comparative analysis and reliability estimates of the video observational and self-report measures. The reliability testing of the observational communication measures was mixed with the individual categories of linguistic and relational statements not meeting the acceptable level of internal consistency (i.e., minimum of 0.75); however, overall PCDC statements (linguistic plus relational statement combined) demonstrated acceptable internal consistency. All three self-report measures (i.e., Provider Interaction Comfort Scale, Personal Accomplishment scale, and Mutuality Scale) demonstrated high internal consistency.

**Table 4.** Paired Samples Testing Summary (unadjusted change over time).

	Pre-intervention Mean (±SD)	Post-intervention Mean (±SD)	t-value	Sig. (2-tailed) *p*-value	Reliability Testing Pre/Post^a^
Video observation measures					
Number of care aide statements	130.4 (68.15)	139.6 (61.05)	−0.515	.618	--
Number of care aide statements coded	88.27 (48.80)	116.5 (52.65)	−2.160	.056	--
Number of resident statements	59.36 (46.31)	71.18 (48.90)	−1.73	.114	--
Linguistic skills					
Sub-total reciprocity statements	47.36 (27.25)	59.36 (29.34)	−2.174	.055	.36/.36
Number of reciprocity categories used	3.82 (0.75)	4.27 (0.65)	−1.838	.096	--
Sub-total clarity statements	16.09 (10.30)	20.55 (10.17)	−1.536	.155	.49/.06
Number of clarity categories used	3.64 (1.12)	4.00 (1.34)	−0.803	.441	--
Sub-total continuity statements	0.00 (0–12)^b^	5.00 (4.86)	--	.014	.57/.45
Number of continuity categories used	0.82 (0.98)	1.64 (0.92)	−2.324	.042	--
Total linguistic statements	66.00 (36.71)	84.64 (40.84)	−2.249	.048	.71/.60
Number of relational statements	49.00 (23.38)	66.64 (30.0)	−1.862	.092	.72/.72
Number of uncategorized statements	42.00 (26.32)	23.09 (12.62)	2.584	.027	--
Overall PCDC statements	114.8 (59.49)	151.27 (67.1)	−2.077	.065	.80/.79
^c^Self-report measures					
Providers interaction comfort scale	45.0 (9.13)	50.18 (5.44)^d^	15.862	<.001	.88/.85
Personal accomplishment score	42.09 (6.76)	41.73 (3.58)	0.188	.855	.97/.86
Mutuality scale	41.09 (10.16)	42.82 (7.73)	−0.699	.501	.92/.87
Relationship closeness VAS (Global rating)	63.45 (27.05)	69.0 (28.54)^d^	7.544	<.001	--
Self-reflection at work VAS (Global rating)	61.73 (33.82)	88.36 (12.32)	−2.435	.035	--

*N* = 11; significance level of 0.1; All paired samples with normal distributions were compared using the paired samples t-test with one exception indicated by^b^.

^a^Cronbach’s alpha.

^b^Non-normal distribution; median (range) and related-samples Wilcoxon Signed-Rank results reported.

^c^Providers Interaction Comfort Scale range 0–60; Personal Accomplishment Score range 0–48; Mutuality Scale range 0–60; Relationship Closeness VAS - global rating range 0–100; Self-Reflection at Work VAS - global rating range 0–100.

^d^Normal distribution with Log10 transformation.

#### Communication observational measures

Observations from the pre- and post-intervention videos were used to assess whether the communication intervention improved the care aides’ use of linguistic and relational elements of PCDC. Overall, 2970 care aide statements were included in the PCDC coding and analysis: 1537 pre-intervention and 1533 post-intervention statements.

In respect to linguistic statements, there was a significant increase (i.e., *p* < 0.1) in reciprocity statements (t = −2.174, *p* = .055) and the number of reciprocity categories (t = −1.838, *p* = 0.96) used by the care aide participants. There was also a significant increase in continuity statements (*p* = .014) and number of continuity categories (t = −2.324, *p* = .042) compared to pre-intervention measures. There was not a significant change in the number of clarity/coherence statements or categories used. Overall, there was a significant increase in the total number of linguistic statements (t = −2.249; *p* = .048).

There was also a significant increase in the total number of relational PCDC statements used by the care aides (t = −1.862; *p* = .092). When linguistic and relational statements were combined, there was a significant increase in PCDC behaviours overall (t = −2.077; *p* = .065).

#### Care aide self-report measures

Responses from the pre- and post-intervention questionnaires were used to examine perceived competence and confidence in PCDC [i.e., Providers Interaction Comfort Score (PICS)], relationship satisfaction [i.e., Personal Accomplishment scale (PA)] and relationship closeness [i.e., Mutuality Scale (MS) and global rating Provider Close VAS] with the resident partner, and self-reflection at work (i.e., Self-reflection VAS). There was a significant increase in the PICS scores (t = 15.862; *p* < .001). There was a significant increase in global reports of relationship closeness with the resident (t = 7.544; *p* < .001) and global reports of self-reflection at work to understand the resident’s situation (t = −2.435; p .035). There was not a significant change in MS or PA scores.

### Covariate analysis

The detailed results of the analysis of covariate influences are available from the first author. Variables that appeared to have the largest impact on the PCDC observational outcomes were: care aide level of education, current position, shift worked, country of birth, pre-video assistance, resident age, and ISE score. Variables that appeared to have the greatest influence on the care aides’ self-reported measures of relationship closeness, reflection at work, and provider interaction comfort were: care aide level of education, gender, first language, number of shifts worked in the past two weeks, years worked in LTC, years worked as a care aide, resident ISE score, and type of dementia.

## Discussion

The results of this pilot study suggest that the PCDC intervention incorporating VF had a positive impact on aspects of care aides’ person-centred dementia communication skills and closeness of the relationship with the resident. There was an observed increase in the use of linguistic reciprocity and continuity communication skills, as well as relational statements. Care aides’ reports of competence and confidence in PCDC communication skills, relationship closeness with the resident, and self-reflection at work also increased after the intervention.

### Communication outcomes

Study findings in respect to self-reported increases in dementia communication competence and confidence adds support to the existing body of literature. Similar outcomes have been documented regarding nurses’ (McGilton et al., 2010) and care aides’ (Passalacqua & Harwood, 2012; Williams et al., 2016) responses to person-centred communication interventions. As many residents in LTC experience dementia, it is imperative that communication interventions developed for care aides support knowledge enhancement, as well as boost confidence and willingness to utilize learned skills and engage residents in conversation (Williams et al., 2016).

Increases in the use of linguistic approaches were seen across two of the three categories of linguistic skills (i.e., reciprocity and continuity) and aligns with existing findings in the literature (Gerritsen et al., 2018; Noordman et al., 2014; van Weert et al., 2011). Enhanced use of relational approaches was also a suggested outcome of the study. The increased use of relational statements by the care aides suggests that the person-centred care principles of valuing the person experiencing dementia, individualizing care, understanding the perspective of the person, and providing a supportive social environment (Brooker, 2007; Kitwood, 1997) may be positively impacted by the intervention.

To aid learning of the multiple PCDC strategies, participants noted that the breakdown of the linguistic and relational elements into manageable pieces was particularly helpful. Additionally, the care aides reported that the memory-aid of the communication strategies was an effective learning and retention approach. Participants also indicated that the self-reflective components of the intervention (e.g., self-reflective activities, role play and viewing/commenting on own video performance) had a key impact on their learning and resultant change in communication behaviours.

### Relational outcomes

The study’s findings suggest an improved quality of the care aide-resident relationship and increased feelings of closeness with the resident, which align with previous intervention study findings (Coleman & Medvene, 2013; Damen, et al., 2011; Gerritsen et al., 2018). Interventions that support positive communication outcomes have included the use of relational strategies. The development and maintenance of a close relationship are believed to facilitate ‘good’ and meaningful communication between care providers and individuals with dementia (Alsawy et al., 2017).

One aim of the intervention was to foster self-reflection and perspective-taking to raise awareness and stimulate behaviour change in respect to PCDC. A suggestive finding in the study was increased reports of self-reflection of care aides’ work-related actions and behaviours. Emerging research in this and other health sciences fields has begun to delineate the relationship between self-reflection and perspective-taking and examine how these processes may be effectively fostered within care provider education and interventions. Research suggests that self-reflection and perspective-taking are interdependent concepts that share similar outcomes (e.g., increased empathic accuracy in understanding another’s thoughts, feelings, and behaviour) but entail different areas of attention (i.e., self-focus vs. other-focus respectively). It is felt that self-reflection, or thinking upon one’s thoughts, feelings, behaviours, and past experiences, is positively correlated with the ability to take the perspective of others, suggesting that a balanced approach to awareness of the self and the other’s viewpoint are necessary components of perspective-taking (Gerace et al., 2017). When one is aware of one’s own biases, preferences, beliefs, thoughts, or feelings, one can better control them from colouring one’s inferences of another person’s thoughts and feelings on the situation.

### Covariate influences

In this study, care aide variables that had strong effects across outcome measures were current position (e.g., full-time/part-time), amount of work experience as a care aide, and time spent working within the LTC context. These factors could result in more frequent opportunities to interact with residents and in turn impact communication competency and confidence in conversing with residents experiencing dementia.

The resident factor that appeared to have the broadest covariate influence in this study was the level of social engagement, i.e., ISE score. A novel finding, it is hypothesized that the resident’s level of and comfort with social engagement, could impact a care provider’s opportunities and success with communication attempts over time.

### Limitations

Causality of the effects of the intervention cannot be established within the study design and sampling approach. The use of a small convenience sample from one LTC home poses limitations to generalizability of findings. Statistical limitations are also noted in relation to the small sample size. Due to the use of a significance level of *p* < 0.1 for the quantitative analysis, pre- and post-intervention changes in outcomes should be considered suggestive findings. The analysis was largely focused on the linguistic and relational elements of PCDC; thus, other forms of communication analysis such as behavioral, paralinguistic, emotional tone and content analysis of the dialogue (Williams et al., 2018) was not undertaken.

### Future Implications

The potential for VF as a learning technique to enhance self-reflection in care aides is a significant consideration in respect to staff knowledge development and behaviour change. Self-reflection is a process theoretically posited to stimulate self-evaluation with resultant enhancements of cognitive and behavioural performance, as well as heightened self-responsibility (Gerace et al. 2017). Providing this learning opportunity to care aides working in LTC may provide a means to bolster the impact and sustainability of traditional learning techniques.

Future research implications include conducting additional testing of the intervention within a larger study using a comparative study group and including residents who experience severe dementia. Research opportunities also present in relation to refining measurement of PCDC elements and VF outcomes. Based on the findings of this study, further exploration of reliable measures of relationship closeness and self-reflection outcomes is warranted.

## Conclusion

This pilot study suggests that the educational and VF intervention shows promise as an effective means to promote PCDC behaviours in care aides working in LTC when communicating with residents with mild to moderate dementia. The findings imply that the intervention fostered an increased awareness of person-centred approaches within the care aide participants. Facilitating the shift from ‘thinking’ to ‘doing’ is the desired goal of professional development efforts but is often difficult to achieve using traditional training approaches. These study results support undertaking a larger study to assess intervention effectiveness more fulsomely.

## Supplemental Material

Supplemental Material - Connecting Through Conversation: A Novel Video-Feedback Intervention to Enhance Long-Term Care Aides’ Person-Centred Dementia Communication Click here for additional data file. Supplemental Material for Connecting Through Conversation: A Novel Video-Feedback Intervention to Enhance Long-Term Care Aides’ Person-Centred Dementia Communication by Deanne J. O’Rourke, Michelle M. Lobchuk, Genevieve N. Thompson, and Christina Lengyel in Gerontology and Geriatric Medicine.
