# Supplementary material for: Connecting Through Conversation: A Novel Video-Feedback Intervention to Enhance Long-Term Care Aides’ Person-Centred Dementia Communication
Source: Gerontol Geriatr Med. 2022 May 14;8:23337214221101266. doi: 10.1177/23337214221101266 (PMC9109488; doi:10.1177/23337214221101266)
Supplement: Supplemental Material - Connecting Through Conversation: A Novel Video-Feedback Intervention to Enhance Long-Term Care Aides’ Person-Centred Dementia Communication [file sj-pdf-1-ggm-10.1177_23337214221101266.pdf]

Supplemental Materials

1. Study Timeline
2. Person-Centred Dementia Communication Education Session Plan
3. Linguistic Dementia Communication Categories and Coding System
4. Relational Dementia Communication Categories and Coding System
5. Steps Taken to Address Coding Rigor of Video Interactions

1. **Study Timeline**

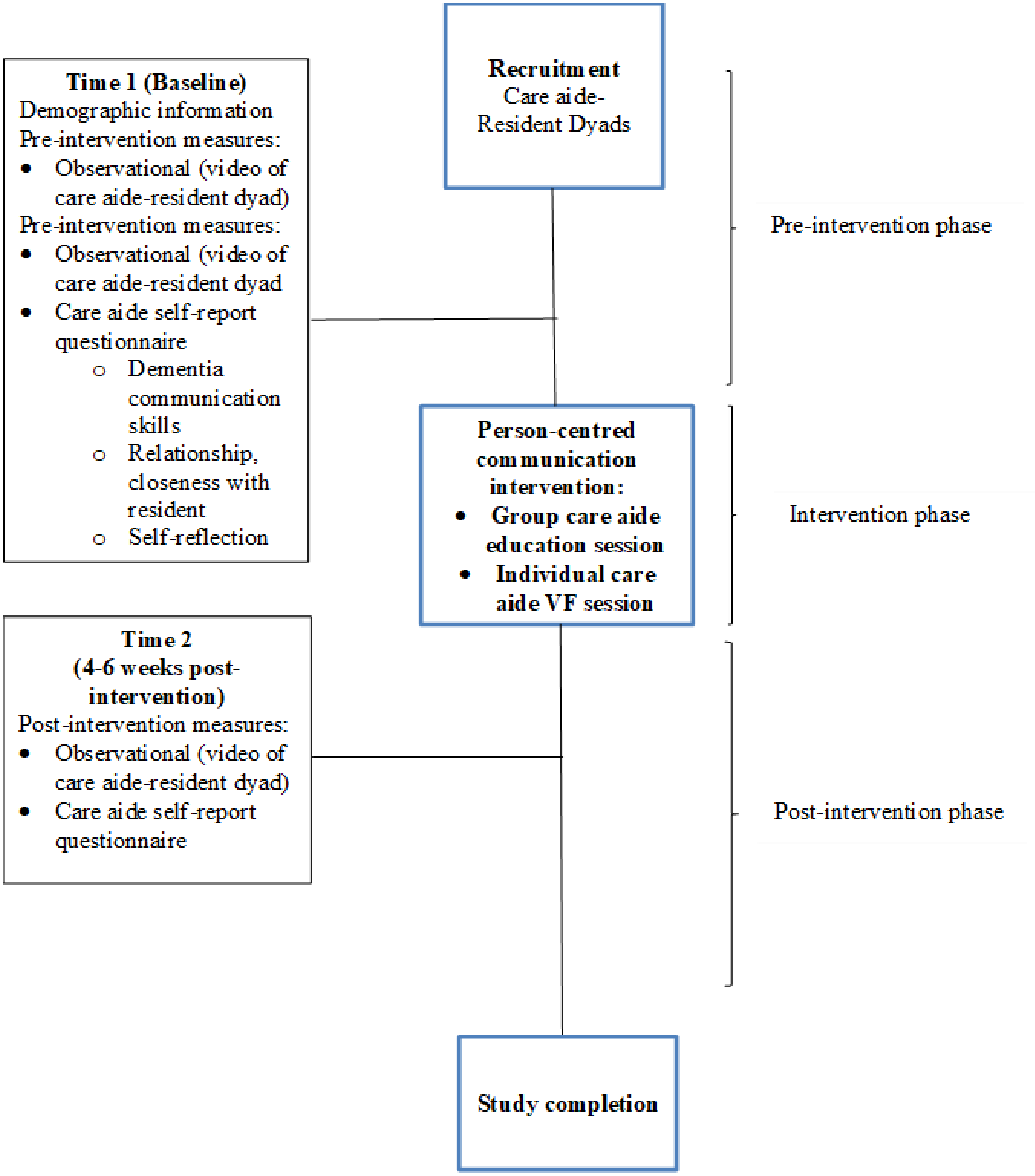

2. Person-Centred Dementia Communication Session Plan (3 hours)

Topic	Materials	Timeframe
Introduction/Goals for the Session	Slide deck	10 min
Overview of Communication<br>- Purpose<br>- Component of good communication<br>- communication challenges<br>- Communication in Dementia	Slide deck	20 min
Person-Centred Dementia Communication<br>- Overall framework<br>- Relational components<br>o VIPS (Person-Centred Care) Framework<br>o How to talk in person-centred ways (4 communication approaches)<br>o Video and role play	- Slide deck with video links<br>- Person-Centred Dementia Communication Checklist handout<br>- Person-Centred Communication Practice (Role Play) handout	60 min
Break		15 min
Person-Centred Dementia Communication (continued – 60 min)<br>• Language-based Dementia Communication Skills<br>o Three conversation goals and associated strategies<br>o Practice activity and discussion – Part 1 and 2<br>• Non-verbal communication	- Slide Deck<br>- Person-Centred Dementia Communication Checklist handout<br>- Dementia Communication (Language) Skills Practice handout	60 min
Reflective Learning/Practice	- Questions for Reflection and Learning handout<br>- Take-home Resident Reflection Activity	10 min
Next steps/wrap-up		5 min

Note: Educational resources and materials are available by contacting the first author.

3. Linguistic Dementia Communication Categories and Coding System

Linguistic Strategy	Examples
**Communication Goal: Reciprocity**	
**1. Greeting**	"Good morning, Sarah."
**2. Completion of turns; giving time for the person to respond; do not interrupt**	Pauses in conversation to allow time to respond.
**3. Open-ended questions that rely on semantic memory, not episodic memory**	"How are you feeling now?"<br>"What do you like about the children's singing?"
**4. Choice questions that rely on semantic memory, not episodic memory**	"What sandwich would you like, beef or egg salad?"
**5. Yes/No questions that rely on semantic memory, not episodic memory**	"Are you hungry?"
**6. Affirmation: statements that indicate agreement, acknowledge feelings or are used with requests/instructions**	"Yes", "Mm-hmm", "I understand."<br>"I know you don't like it when (other resident) is calling out for help. Just hold on a bit as the staff are helping her now."
**7. Politeness to address resistiveness**	"Please don't hit my hand when I try to help you. Do you hurt anywhere right now?"
**Communication Goal: Clarity/Coherence**	
**8. Announce intent clearly; inform about a topic change**	"Now let's talk about…"
**9. Confirm understanding through restating what the resident said, summary of prior talk**	"Let me see if I understand (paraphrase what resident said)…"
**10. Confirm understanding by asking for clarification**	"Are you saying that you would like to…?"
**11. Statements that inform what is misunderstood**	"I don't understand what ___ means."
**12. Rephrase to add clarity to a statement**	Staff: "Would you like to go listen to the school choir?"<br>Resident: "The what?"<br>Staff: "Would you like to go listen to the children sing?"
**13. Verbatim repetition**	Repeating the same verbal statement
**14. Ask the other person to repeat what they said**	"Pardon me?"<br>"Can you repeat that?"

Linguistic Strategy	Examples
**15. Right-branching sentences; avoid left-branching sentences**	Right-branching: "You need to put on your coat before going outside."<br>Left-branching: "Before going outside, you need to put on your coat."
**16. Place modifiers after nouns**	"Do you want juice, apple or orange?"
**17. Place modifiers after verbs**	"Walk slowly with me."
**Communication Goal: Continuity**	
**18. Unfinished sentences that the resident is encouraged to complete**	"Your favorite TV show is _____?"
**19. Matching comment**<br>**20. Matching association; offer one's opinion or information about a personal experience**	Resident: "I like the Family Feud."<br>Staff: "You like The Family Feud? (matching comment) My favorite game show has always been The Price is Right. I remember watching Bob Barker as host when I was a kid (matching association)."
**21. Newsmarks: emphasis on the noteworthiness of the resident's prior statement**	"Really?"<br>"Wow, that's great!"<br>"Oh my!"

(Adapted from Savundranayagam & Moore-Nielsen , 2015)

4. Relational Dementia Communication Categories and Coding System

Relational Strategy	Examples
**Recognition**	
**1. To acknowledge the resident as a person, known by name, affirmed in a unique way (e.g. greeting, listening, eye contact)**	"Hello, Mrs. Smith, <*recognition*>. Are you ready for lunch?"<br>"Good morning, John <*recognition*>. How did you sleep?"
**2. Use of Biographical statements. Code when the HCA refers to something about the resident's family, life, or day. This category is coded *by topic*, not by individual statement. Do not code general statements such as statements about the current weather situation.**	"I saw your sister yesterday in the mall."<br>"Has your husband been to visit lately?"<br>"How many kids do you have?"<br>"Where did you grow up?"
**Negotiation**	
**1. To consult about the resident's preferences, desires, needs.**<br>• **Much negotiation takes place over simple everyday issues, such as whether a person feels ready to get up, or have a meal, etc.**<br>• **Negotiation gives a sense of control to a person with dementia**	Example 1: Mrs. Johnson: "I, uh ... I can't find my place. Oh ... here it is!"<br>Staff: "Actually, your table is over there (pointing). Would you like to walk over together before the meals are served?" <*negotiation*><br>Example 2: Staff: "That was a nice bit of fresh air, wasn't it? I'm ready for my dinner now; would you like to join me?" <*negotiation*>
**Validation**	
**1. To acknowledge the reality of a person's emotions/feelings, and give a response on the feeling level**<br>**2. To appreciate and respond to the desire or need that a person may be expressing; to help if necessary, to convert it to an intention**<br>**3. To use empathy and gain some sense of what a person may be experiencing**<br>**4. To understand a person's definition of the situation**	Example 1: Mrs. Johnson: (eagerly) "Why don't I help set the uhm ... tables – that way ... uh ... lunch'll arrive sooner!" (reaches for the butter knives)<br>Staff: "Mrs. Johnson, that'd be really helpful! <*validation*> We can set the tables together and soon have things under control. <*validation*><br>Example 2: Mr. Lawton: "Where's Mary? Isn't she supposed to be here?"<br>Staff: "Mary will be at work now, and she knows you're here. You have managed well this morning since you've been worried about Mary." <*validation*>

Relational Strategy	Examples
**5. To respond sensitively to any signs that a person's definition of the situation is changing, and to move with any changes that occur**	
**Facilitation/Collaboration**	
**1. To work together**<br>**2. To involve the person's initiative and abilities in a shared task, with a definite aim in view.**<br>**3. To enable a person to do what otherwise he/she wouldn't be able to do by themselves by providing the missing parts of the action**<br>**4. To enable interaction to get started, to amplify it and to help the person gradually fill it out with meaning**<br>**5. To enable a person to sustain his or her action; to keep it from falling into the void because of memory failure**<br>**6. To be ready either to initiate or respond to the resident; neither rushing in too quickly, nor holding back for too long.**<br>**7. To enable the use of remaining abilities by requesting that the resident perform an activity of daily living.**<br>**8. To fill gaps in meaning (note: explaining the task to the resident is only facilitation if it is filling a gap in understanding and the resident prompted the explanation.**	Example 1: Mrs. Smith:(wanders into the dining room) "Have you seen ... have you seen it?" Staff: "What is it you're looking for, Mrs. Smith? Can I help? *<facilitation> Tell me what it is and we can look for it together. <facilitation>*<br>Example 2: Mrs. Rogers: "She knew I didn't want to go to bingo, and instead we uhh..."<br>Staff: "What happened then?" *<facilitation>*
∗Note: to distinguish between validation and facilitation, statements that are more feeling-oriented should be categorized under validation and those that are more action-oriented should be categorized under facilitation.	

(Savundranayagam, 2014)

5. Steps Taken to Address Coding Rigor of Video Interactions

The first author and a trained research assistant independently coded care aide statements from transcriptions of pre-/post-intervention video observations utilizing the described coding systems above. Transcripts were analyzed in conjunction with viewing the original videos to confirm the intent and purpose of statements.

1. All videos were transcribed into separate typed records by an independent transcriptionist with each statement in the transcript referenced by a time stamp (minute:second).
2. The transcripts were reviewed and compared to the videos for accuracy.
3. The research assistant received general training from the first author on the PCDC coding scheme. The second author was also trained in the coding system to act as an independent opinion if coding agreement could not be reached. Coding practice and discussion between the first author and research assistant proceeded on one study-related video/transcript until 100% agreement was reached.
4. The entire length of each of the remaining 20 videos were then independently coded by the first author and research assistant in the following manner:
   i) Each care aide statement in the video/transcript was reviewed sequentially for evidence of linguist and/or relational PCDC strategies and noted on the transcript adjacent to the associated time stamp;
   ii) If a statement exhibited both linguist and relational strategies, it was coded as having two code counts (one for linguist and one for relational);
   iii) If a statement did not exhibit any strategy in the PCDC coding system, it was coded as ‘Uncategorized;

iv) The first author and research assistant met either in person or via phone to discuss the results of the independent coding until 100% agreement was reached on all transcripts;

v) At the completion of the video coding, the totals were calculated and recorded on the coding sheet for the following:

(1) Number of care aide statements;

(2) Number of care aide statements coded;

(3) Number of uncategorized statements;

(4) Number of resident statements;

(5) Number of statements for each of the 21 linguist strategies;

(6) Sub-total of linguist statements for each of the three communication categories (i.e., reciprocity, clarity, and continuity statements);

(7) Number of linguist strategy types used in each of the three communication goal categories;

(8) Total number of linguist statements;

(9) Number of statements for each of the four relational strategies;

(10) Total number of relational statements; and

(11) Total number of PCDC statements (linguist plus relational).
